# Stakeholder perspectives on antenatal depression and the potential for psychological intervention in rural Ethiopia: a qualitative study

**DOI:** 10.1186/s12884-020-03069-6

**Published:** 2020-06-22

**Authors:** Tesera Bitew, Roxanne Keynejad, Simone Honikman, Katherine Sorsdahl, Bronwyn Myers, Abebaw Fekadu, Charlotte Hanlon

**Affiliations:** 1 Department of Psychology, Debre Markos University, Institute of Educational and Behavioural Sciences, Debre Markos, Ethiopia; 2 Department of Psychiatry, Addis Ababa University, College of Health Sciences, School of Medicine, Addis Ababa, Ethiopia; 3 Section of Women’s Mental Health, Institute of Psychiatry, Psychology & Neuroscience, King’s College London, London, UK; 4 Perinatal Mental Health Project, Department of Psychiatry and Mental Health, University of Cape Town, Cape Town, South Africa; 5 Alan J. Fisher Centre for Public Mental Health, Department of Psychiatry and Mental Health, University of Cape Town, Cape Town, South Africa; 6 Alcohol, Tobacco and Other Drug Research Unit, South African Medical Research Council, Cape Town, South Africa; 7 Department of Psychiatry & Mental Health, University of Cape Town, Cape Town, South Africa; 8 Addis Ababa University, College of Health Sciences, School of Medicine, Department of Psychiatry, WHO Collaborating Centre in Mental Health Research and Capacity-Building, Addis Ababa, Ethiopia; 9 Centre for Innovative Drug Development and Therapeutic Trials for Africa (CDT-Africa), College of Health Sciences, Addis Ababa University, Addis Ababa, Ethiopia; 10 Global Health & Infection Department, Brighton and Sussex Medical School, Brighton, UK; 11 King’s College London, Institute of Psychiatry, Psychology and Neuroscience, Department of Psychological Medicine, Centre for Affective Disorders, London, UK; 12 King’s College London, Institute of Psychiatry, Psychology and Neuroscience, Health Service and Population Research Department, Centre for Global Mental Health, London, UK

**Keywords:** Antenatal depressive symptoms, Conceptualisation, Acceptability, Qualitative study, Ethiopia

## Abstract

**Background:**

Psychological interventions for antenatal depression are an integral part of evidence-based care but need to be contextualised for respective sociocultural settings. In this study, we aimed to understand women and healthcare workers’ (HCWs) perspectives of antenatal depression, their treatment preferences and potential acceptability and feasibility of psychological interventions in the rural Ethiopian context.

**Methods:**

In-depth interviews were conducted with women who had previously scored above the locally validated cut-off (five or more) on the Patient Health Questionnaire during pregnancy (*n* = 8), primary healthcare workers (HCWs; nurses, midwives and health officers) (*n* = 8) and community-based health extension workers (*n* = 7). Translated interview transcripts were analysed using thematic analysis.

**Results:**

Women expressed their distress largely through somatic complaints, such as a headache and feeling weak. Facility and community-based HCWs suspected antenatal depression when women reported reduced appetite, sleep problems, difficulty bonding with the baby, or if they refused to breast-feed or were poorly engaged with antenatal care. Both women and HCWs perceived depression as a reaction (“thinking too much”) to social adversities such as poverty, marital conflict, perinatal complications and losses. Depressive symptoms and social adversities were often attributed to spiritual causes. Women awaited God’s will in isolation at home or talked to neighbours as coping mechanisms. HCWs’ motivation to provide help, the availability of integrated primary mental health care and a culture among women of seeking advice were potential facilitators for acceptability of a psychological intervention. Fears of being seen publicly during pregnancy, domestic and farm workload and staff shortages in primary healthcare were potential barriers to acceptability of the intervention. Antenatal care providers such as midwives were considered best placed to deliver interventions, given their close interaction with women during pregnancy.

**Conclusions:**

Women and HCWs in rural Ethiopia linked depressive symptoms in pregnancy with social adversities, suggesting that interventions which help women cope with real-world difficulties may be acceptable. Intervention design should accommodate the identified facilitators and barriers to implementation.

## Background

Antenatal depression is a public health challenge, affecting 10 to 20% of pregnant women [1, 2]. Prevalence estimates for antenatal depression are higher in low- and middle-income countries (LMICs) compared to high-income countries (HICs) [3–5]. Antenatal depression is associated with reduced dietary intake among pregnant women [6–8], delayed initiation of breast-feeding [9], reduced infant bonding and infant under-nutrition [8, 10, 11]. When untreated, antenatal depression may persist into the postnatal period [12, 13] and is associated with a range of adverse consequences, including: impaired functioning [14–16], increased risk of physical perinatal complications [17], higher emergency healthcare costs [18, 19] and mortality due to suicide [20]. Evidence-based interventions in the antenatal period can reduce the adverse effects of antenatal depression [21] and decrease the risk of persistence into the postnatal period [11, 12].

Both ante- and postnatal depression in LMICs are linked with high levels of social adversity, including poverty and marital problems [22, 23]. In a previous qualitative study in Ethiopia [24], women attributed antenatal emotional distress to worry about exacerbation of poverty and marital problems, fear of death, perceived vulnerability to physical and supernatural misfortune and concern about the forthcoming delivery [24]. Despite amenability to psychological interventions, ante- and postnatal depression in LMICs are under-detected and evidence-based treatments are limited [18]. The treatment gap for women with postnatal depression with respect to accessing evidence-based mental health care was found to be 95% in a rural Ethiopia district [19]. Indeed, women with depressive symptoms mainly received help from informal sources like husbands, friends and parents [19] and relied on religious and spiritual coping strategies [25].

A number of manualized psychological interventions, such as cognitive behavioral therapy [26–28], interpersonal therapy and psycho-education [28, 29], have been shown to be effective for antenatal depression. These interventions include both specific and non-specific elements [29] and have been delivered successfully by non-specialists in a task-shared model in primary healthcare settings [30]. Most of these intervention studies were conducted in middle-income countries; we are not aware of any studies from rural areas of low-income African countries [27].

Given sociocultural and health system differences, psychological interventions must be adapted to fit the local context. A key aspect of ensuring that an intervention fits the local context relates to the extent it maps onto the explanatory models of the problem that are held by both affected persons and potential intervention providers [31]. The concept of the explanatory model was developed by Arthur Kleinman [32] and encompasses the person’s causal attribution and labeling of the problem, perception of severity and prognosis, as well as coping strategies, preferred help-seeking and interventions [32]. Explanatory models combine with structural factors, in particular socioeconomic status, to influence the way in which women recognise and communicate symptoms of depression, the coping strategies they employ [22] and how they access and engage with treatments [33]. Similarly, the explanatory models of healthcare providers may affect their thinking, attitude and behaviours in relation to antenatal depression, which in turn can influence their detection of mental health problems and factors such as therapeutic rapport during delivery of interventions [34].

Nevertheless, the perspectives of Ethiopian women who have experienced mental health problems during pregnancy, and of HCWs who might deliver psychological interventions for antenatal depression, are not well understood. This study, therefore, aimed to explore service users’ and service providers’ perspectives of antenatal depression and the potential for future psychological interventions for antenatal depression in the Ethiopian context. The results of this study will inform future adaptation of a culturally relevant psychological intervention for antenatal depression in this setting.

## Methods

### Design

We conducted a qualitative study comprising in-depth interviews with key stakeholders in relation to antenatal depression in rural Ethiopia.

### Setting

The study was conducted in Sodo district, south central Ethiopia, which encompasses 58 sub-districts (54 rural and four urban). There are eight primary healthcare centres (PHCs), each about 25,000 population, and one district level primary hospital. The facility-based PHC workers (nurses, midwives and health officers (BSc degree level healthcare workers trained both in clinical and preventive activities) provide antenatal, delivery and postnatal care. Ethiopian women are expected to attend at least four antenatal care appointments, with at least their first and last antenatal care appointments at the PHC facility. Women without complications can attend the remaining antenatal care visits in health posts. Each PHC facility is associated with five ‘health posts’ which are grassroots level community-based healthcare facilities, each staffed by two health extension workers (HEWs). HEWs are community-based healthcare workers responsible for core preventive and public health promotion activities, including reproductive health, hygiene and maternal health [35]. HEW duties include the identification of pregnant women in the community, linking women to facility-based antenatal care and providing some antenatal care at the community level. Over the past 8 years, the Programme for Improving Mental Health carE (PRIME) team [36] worked with district health office planners and local stakeholders to implement a mental health care plan based on task-shared care delivered in primary and maternal health care settings in Sodo district. In the absence of a contextually adapted psychological therapy, the model of task-shared maternal mental health care implemented in PRIME was limited to psycho-education, basic psychosocial support and antidepressants for women who were more severely unwell.

### Sample recruitment

Women who scored five or more on a locally validated version of the Patient Health Questionnaire (PHQ-9) during pregnancy as part of our previous study [37] (conducted between September 2014 to June 2015) [38], were invited by project workers to participate in an interview. Interviews took place during December 2017. Healthcare workers engaged in maternal care, PHC facility-based health workers and health extension workers, were identified and invited to participate through key informants (district health office representatives). We used purposive sampling to identify clinical staff with varying qualifications, levels of experience and who were based in rural and urban health facilities. Recruitment of participants continued until no new perspectives arose, i.e. until theoretical saturation was attained.

### Procedures

All interviews were conducted in the most conveniently located health centre for the participant. Research assistants (females for women and PHC workers; and a male for HEWs) with at least a master’s level degree and experience with qualitative research conducted all interviews in Amharic, the official language of Ethiopia. Interview topic guides were informed by Kleinman’s explanatory model interview [32, 39]. Accordingly, the interview topic guide for women explored: their experience, description and explanations of emotional difficulties in pregnancy; impact of emotional difficulties; coping strategies; treatment preferences; their expectations of HCWs, family and traditional healthcare providers; and barriers and facilitators in relation to accessing psychosocial support (supplementary file 1). We used the term ‘emotional difficulties’ to avoid imposing a medical conceptualisation of their experiences and because the term ‘depression’ is not familiar to women in the study site. The interview topic guide for facility-based PHC providers explored: emotional and social problems faced by women in pregnancy, pregnant women’s conceptualisation of depression, types of support provided to women with emotional problems, identifying women with depression, acceptability of psychological interventions and suggestions for how such interventions might be adapted and implemented (supplementary file 2). The interview topic guide for HEWs explored: their role in helping pregnant women seek healthcare, barriers to and motivators of women’s help seeking, responses to women not attending antenatal care, problems women face during pregnancy, ways to help women access antenatal care, presentations of emotional problems and their perception of women’s treatment preferences (supplementary file 3). Participants were reimbursed for their transportation. The interviews lasted between 40 and 82 min. All interviews were audio-recorded, with the permission of the participants.

### Data analysis

Audio-recorded interviews were transcribed verbatim and translated into English. The first two transcripts were coded independently by two reviewers (TB generated 58 codes and RK 53) using Open Code qualitative analysis software [40]. Discrepancies were discussed to reach consensus on the naming, merging and creation of new codes, yielding a final 55 codes which were then condensed after discussion with the senior author (CH) to 36 (see: Supplementary file 4). One of the reviewers (TB) then coded the remaining transcripts using this final set of agreed upon codes. No further codes emerged from the remaining data. Thematic analysis was employed, where the codes were synthesized into four themes and then into sub-themes through discussion between CH and TB. Each transcript was then summarized in a spreadsheet based on themes and sub-themes (see: Supplementary Table). However, the third and fourth themes overlapped substantially, so were merged.

## Results

### Participant characteristics

The 23 interviewees comprised eight women who had previously screened positive for antenatal depressive symptoms, eight facility-based PHC providers (‘providers’) and seven HEWs (See Table 1).

**Table 1:** Participant characteristics

Participants	N	Age (years)	Education
Women with a history of antenatal depressive symptoms	8	23–35	Ranging from non-literate to seventh grade of high school
PHC providers (5 females and 3 males):			
Midwives	5	24–29	Ranging from Diploma to BSc
Nurses and Health officers	3	24–29	BSc Degree
Health Extension Workers	7	23–34	Level 4 (two years training)
Total number of participants	23		

The two major themes which emerged from thematic analysis of all narratives were: 1) concepts and contexts of antenatal depression and 2) acceptability and feasibility of brief psychological interventions.

### Concepts and contexts of antenatal depression

Three subthemes were identified within this theme: 1) manifestations of antenatal depression, 2) conceptualisation of antenatal depression and 3) existing coping mechanisms for depressive symptoms.

#### Manifestations of antenatal depression

Women commonly described emotional problems through somatic complaints: feeling ‘*worn out’, ‘like feeling of burning on the forehead’*, ‘*my head is tense’ and ‘headache’.*

*“I feel like my head is tense … then I get stressed. I was not able to sleep. When I have a headache, then I physically get exhausted.”* Woman, 005.

*“I didn’t sleep a lot, but I feel tired. When I sit in one place, I want to sit for a long time and [I am] unable to get up quickly”* Woman 006.

However, this was not reflected in the HEWs’ descriptions, despite their closeness to the community. Women presented their emotional problems to HEWs as feeling of tension arising from situational events like conflict in their marriage or with their step-children. For example, a woman’s presentation of the symptoms to a HEW: “*I get tense when I hear something, or when I hear children crying at home, or when he [husband] speaks* …. *I couldn’t afford tension*”, HEW 008.

In some women, depressive symptoms were linked to psychological manifestations like a wish to die (“*Sometimes I wish God would take my life.*” Woman 002; “*Sometimes when I got stressed so much, I felt like hanging myself.” Woman 008*) or to the wish to isolate themselves from other people (“*I don’t feel good when talking with people.”* Woman, 005; “*I don’t know the reason why, but I want to live alone.”* Woman 006).

Both facility-based providers and HEWs tended to characterise antenatal depression in terms of observed behaviours ("*the symptoms of depression are seen from their face.*" Provider 002) and reported symptoms, like refusing to breastfeed, social withdrawal, reduced appetite, poor sleep (*"The woman feels sleepy [tired], she has low appetite... she lacks interest and yawns …*" Provider 001), reduced self-care, tiredness and crying. They especially highlighted the difficulty that women with depression have relating with others, manifested in poor engagement with the clinicians. In some instances, HEWs observed women who were not able to access the support they needed: *"they [women] feel that they are not heard by the community,* (HEW 015)*". Indeed, s*ome facility-based providers reported that they ask women about depressive symptoms when they observe poor self-care or weight loss, or if something did not seem to be right such as women crying at home or being silent and unhappy during delivery.

*"When you notice something in their face you will ask them if there is any problem at home. .... they just respond generally.... they do not tell you something specific.... "They get depressed, they cry, sometimes they get tired easily …*. *If there are such symptoms, then I suspect this problem [depression]",* (Provider 004*).*

Facility-based providers also reported cognitive characteristics of emotional problems, such as forgetfulness, ambivalence towards the baby and “*thinking too much*” [“*bizu bemaseb mechenanek*”], anger and ruminating on unattained expectations with feelings of guilt and anxiety as additional manifestations of antenatal depression. HEWs’ and women’s accounts of antenatal depression focused almost entirely on “thinking too much”.

“… *well, I told them [healthcare providers] that I feel mentally stressed...ehh … and they asked me, “What makes you feel stressed”? Then, I replied that, it’s because I think about so many things … ehh … it’s because of the thoughts*”, Woman 001.

#### Conceptualisations of antenatal depression

All types of participants conceptualised antenatal emotional problems as “thinking too much”, which was presented as rumination and a mental overload due to situational challenges such as poverty, physical health and marital conflicts ( “*… first, more than the time they [women] devote for their work, they devote their time thinking and ruminating about ideas or work”, HEW 008).*. “Thinking too much” was conceptualised as a symptom as well as the name of a cluster of problems, associated with sleeplessness, reduced appetite, tiredness and difficulty doing day-to-day tasks, as well as the cause of ongoing emotional problems because of dwelling on unfulfilled expectations.

*“The illness starts when there is a problem. For example, if I get upset with something, when I think about myself, when I couldn’t able to have something that I used to have before … when I think of these things, I feel something. ehh … then, … my mind gets stressed.”* Woman 005

*I used to think what would I eat after giving birth, how am I going to live, I can’t work having my child … ehh … because it’s difficult to take care of the child until he grows up, right? … so, I was very much worried about those things.",* Woman 002

All types of participants reflected that the issues that triggered *“thinking too much”* were related to life difficulties (*“The first thing is my living situation. I used to worry about what I am going to give her [child]. I feel bad so much.”* Woman 007). These difficulties included difficulty improving their livelihood, marital conflict and reproductive health challenges. Women were expected to secure food for their families and improve living standards, in addition to carrying out their household chores and farm obligations, making pregnancy a time of particular anxiety:

*“I am living a very despicable life … ehh … I don’t have a house, I don’t have land … ehh … it’s very difficult to raise these five children. Thus, I started to become stressed when I think of that … ehh … I think of everything, I am living a stressful life.”* Woman 001.

Reproductive health problems that triggered ‘thinking too much’ included: pregnancy complications, grief about perinatal losses, difficulty conceiving, post-operative symptoms in women who had undergone an operative delivery, labour complications, unplanned pregnancy (especially pregnancy before marriage) and childbirth with close birth spacing. These reproductive health problems added to the stress associated with their poor livelihood and challenges of pregnancy.

Lack of compassion among delivery unit staff and appropriate perinatal care were additional challenges perceived as antecedents to thinking too much. For example, one participant recalled a HCW informing her of the death of her newly delivered baby by saying: *“The child is passed, you can’t do anything”.* Staff and women acknowledged the tension between the community’s judgement of women who conceived shortly after a previous pregnancy (“*why is she giving birth like a goat?”)* and husbands’ negative attitudes towards contraception and expectations of having many children. Primiparous women raised worries about effectively nurturing and breastfeeding their first child, including whether their breast milk would suffice.

Another critical contributor that led to “thinking too much”, shared by all groups of participants, was marital conflict. Women reported marital conflict in relation to contraception, disciplining step-children, family illnesses and their husbands’ alcohol use.

*“My husband always nags me. He is always insulting me. ....that is the case and aggravates my illness … If he makes me free, my mind will be free, but he didn’t make me free; due to this I will stress. When the time is going to dark, I will worry because he usually comes drinking alcohol at the night times and I will prepare myself – how to respond to him. The other problem is he didn’t allow me to meet with other people in the area.”* Woman 006*.*

Ultimately, several women attributed everything to the will of the supreme power (“*I asked God what wrong I have done to deserve this and it all got worse …*” Woman 003). The women spoke of the supreme power predetermining all the miseries and joys of their life:

“*If I can work hard to raise him now, he [child] will support me when he grows up, but God didn’t want that to happen, and I lost both of them. My illness is from God, and nobody has done anything to me. [My] husband insults, hits me and drinks.”* Woman 006.

PHC providers reported that women also associated depressive symptoms with Satan or evil spirits or being bewitched (*“um …*. *in postnatal for example, ... a mother is not left alone..., there is another religious saying or it is evil, or demon they will say, … um, during that time, if she has an accident, … she might encounter depression.” Provider 006)* or the society may give it different name*.*

### Existing approaches to cope with distress

PHC providers noted that women did not consider depressive symptoms to be indicative of a mental illness ( “… *since they [women] don’t consider it as an illness, there is less of a chance of visiting health centres … , they spend their lives with the suffering.” Provider 007*). As women understand their symptoms to arise from a supernatural source, it was assumed that they would first seek help for antenatal depression from traditional healers or holy water sites, elders or experienced persons or ‘await God’s will’ in isolation at home despite accepting modern treatment for physical health problems.

*“They mostly choose to sit home alone and cry or go to churches and holy water than discussing the problems with people”,* Provider 002.

However, a few women reported dissatisfaction where religious leaders were not always attentive to their emotions (*“I didn’t go church, .... They don’t hear what I am saying and I don’t hear what they are saying. In fact, I don’t hear their praying”,* Woman 008).

Only a few women used conversation with neighbours and having fun to forget distressing memories associated with their depressive symptoms (“*I fool myself just like babies.”,* Woman 005).

*“I feel better when I have fun and when I chat with people”..*. *“I try to take [myself] out of the situation and try to do other stuff or go to my neighbour’s or friend’s house when I feel like this; that is it”,* Woman 003.

On the other hand, several women did not disclose symptoms to HCWs. Women and HCWs described that women with emotional problems are mostly silent, lacking familiarity with expressing themselves (*“I just went to the clinic … I told nobody at that time … ehh … I was worried by myself”,* Woman 002*)* and struggling to trust and confide in clinicians (“*they come to the facility and take what they want but refuse to discuss their problems”,* Provider 002*).* They noted that some women tell their husbands about depressive symptoms first, whilst others tell neighbours, but most do not tell anyone:

*“I can’t. I was stressed out by saying ‘How could I tell this?’. I did not tell. People just know my condition without me saying ‘I am sick and stressed’. Because I don’t want to stress out another person. I say I will try my best without telling to anyone. That makes me stressed more” … “Most of the time I did not want to bother anyone. Some people come and only tell part of their problem to get pills or some kind of medicine for temporary relief”,* Woman 008.

PHC providers and HEWs reported that they encouraged women to draw on support from their neighbours and husbands in order to address their life problems and emotional difficulties.

### Acceptability and feasibility of psychological intervention

Perspectives on the acceptability and feasibility of potential psychological interventions were grouped into three sub-themes: cultural and structural issues, factors related to detection and referral, and intervention session structuring,

#### Cultural and structural issues

Participants noted that attendance for the intervention might be influenced by some women avoiding being seen in public during pregnancy for fear of evil spirits or social censure, especially when primiparous, frequently pregnant or diagnosed with human immunodeficiency virus (HIV). Culturally, women are restricted from social activities in the final months of pregnancy for fear of being labelled ‘*ayen awuta*’ (‘shameless’) (Provider 003), contributing to social isolation and limiting women’s ability to access treatment and support. Beliefs about how diseases and spirits are encountered outdoors (e.g. through harmful exposure to cold air, rays of sunlight and shady places) also inhibited women from seeking mental healthcare in the perinatal period.

Furthermore, some HCWs noted that in communal cultures like Ethiopia, decisions are shared, or parents may make decisions on a woman’s behalf. Therefore, a woman may not be able to freely attend the health facility for psychological therapy without the consent and approval of her husband. One participant recommended counselling husbands and other family members about the need to provide general support to pregnant women (HEW 010).

Health extension workers and facility-based providers proposed that uptake of psychological interventions would be facilitated by the availability of well-furnished, attractive, accessible maternal healthcare facilities staffed by friendly, motivated, trained staff and integrated into primary healthcare. Most primary healthcare providers asserted that delivering psychological interventions would form part of their responsibilities within routine antenatal care and would not constitute an additional burden. They supported the benefits of psychological interventions for reducing depressive symptoms and the risk of suicide. As a result, several said that that their involvement in providing care which led to a reduction in women’s suffering would be a source of satisfaction:

*“I mean it serves them so that they will not harm themselves because sometimes they go to the extent of suicide when they are in worse condition so, if we can resolve their problems earlier, it will serve to prevent them from getting to their worse state. It gives us satisfaction”,* Provider 003.

Nevertheless, HCWs acknowledged their limited skills in mental healthcare. HEWs identified various challenges, including difficulty detecting antenatal depression, the lack of brief clinical screening tools for depression, insufficient training and the potential for them to become overburdened. One PHC provider reported difficulty communicating about sensitive subjects, such as intimate partner violence and diagnoses such as hepatitis (Provider 002). Staff interviewees suggested coinciding psychological intervention sessions with ANC attendance and assigning specific duties to each HCW to reduce waiting time for appointments. One staff interviewee identified multiple, unintegrated, healthcare strengthening initiatives being implemented in parallel as a barrier to delivering psychological interventions for antenatal depression. Poverty, heavy domestic workloads, social obligations to attend funerals and festivals and fear of being diagnosed with diseases such as hepatitis (*“Mothers fear being diagnosed with another disease”,* HEW 011) were additional barriers to attending for psychological interventions. Further challenges to acceptability included distance and topography between a woman’s home and the health centre and unsupportive husbands (HEW 010). Providing coffee prior to sessions and subsidising transport to attend were suggested as ways of encouraging attendance.

#### Detection and referral

Participants viewed the structure of the existing health system and recent mental health training as an opportunity to promote women’s mental health. Although PHC providers reported feeling confident to ask women about symptoms of depression, most said that they did not conduct a formal assessment using the World Health Organization guidance, despite having been trained to do so, perceiving it as a lengthy process. Only a few HCWs reported experience of detecting antenatal depression (*“I have worked for four and half years and I have encountered one case”,* Provider 007), but even then, the identified symptoms were reported to be mild.

*“the people that come here are usually rural people. We do ask them about it. It doesn’t totally fulfil depression criteria, but it somehow does partly for the mild depression”,* Provider 005.

*“Mild problems like wanting to be alone and cry with their husbands are common. But, no report of severe symptoms”,* Provider 004.

As a result, healthcare workers admitted that depression was likely to be diagnosed if there are other concerns about the woman’s actions, for example if a woman declined to attempt breast feeding:

“*… from my experience if I have many patients, I might not ask this thing. I only ask such questions only if there are not many patients or if I notice something on her body. I haven’t ever asked patients with a prepared question”*, Provider 005.

PHC providers reported that women might be distracted by the many questions that HCWs need to ask about their pregnancy, creating a barrier to disclosing emotional problems (*“After hours of discussion women forget everything when they are asked for reflection. Sometimes they feel they were nagged”,* Provider 001). HCWs noted that the ways that women are asked about their problems influences whether they share their symptoms:

*“For example, asking it as part of the healthcare. Starting from greetings, when we start exchange of ideas about her children, work, etc, she tells us clearly without hiding anything. To raise about this issue [depression] however, there should be another issue to link with or like utilization of family planning which she is following up or which she may also have discontinued. That is something that links with us”,* HEW 008.

One suggested including depression screening questions for all women, in order to have a record of the woman’s emotional wellbeing during pregnancy. PHC providers stressed that detecting depression depended on gaining the trust of the woman, emphasising confidentiality and professional ethics, and asking about social adversities before raising mental health:

*“The providers’ closeness to the mother matters a lot. … Understand needs of women, greet them at beginning, call her name properly, looking their face instead of focusing on paper. … If you are doing something else like writing while she talks, she will not be comfortable”,* Provider 001.

Referral to psychiatric nurses in a nearby hospital was reported (Provider 007) for more severe cases (Providers 004, 006, 007). However, some staff doubted whether women would adhere to referrals.

#### Intervention session structuring (providers, place and sessions)

Most women expressed trust in HCWs to deliver psychological interventions (*“She [woman] will get a big lesson from that advice”,* Woman 007). Women preferred the intervention to be delivered closer to home, by a person perceived as having expertise and being trustworthy, and that the sessions should be aligned with existing ANC visits. They reported that midwives, nurses, HEWs or combinations of these HCWs would be acceptable people to deliver psychological interventions.

*“The woman value advice more than money … They [women] consider health professionals sometimes just next to their Lord. Due to this, they tell us each and everything that they faced”,* Provider 008.

The staff themselves said: *“...mothers really see us as authority figures”,* Provider 001, although one woman had lost trust in them following the death of her baby. Considerations from HCWs included the contribution to existing workloads, with expectations that health centre-based PHC providers may have more time than HEWs to deliver an intervention, and concerns regarding privacy and confidentiality. Although some participants favoured individual or group intervention delivery, most HCWs and women emphasised the benefits of individual sessions for privacy.

## Discussion

In this qualitative study conducted in a rural Ethiopian setting, we explored stakeholders’ conceptualisations of antenatal depression and the potential challenges to, and opportunities for, adaptation, delivery and uptake of perinatal psychological interventions. Although stakeholders agreed that there was a great need for a psychological intervention to address depression during the perinatal period, this study identified several areas that should be considered when adapting an intervention for this cultural context, including explanatory models of depression that emphasize social attribution and somatic manifestation of depression.

Women with a history of antenatal depressive symptoms often described somatic symptoms, such as headache and feeling weak. Participating HCWs spoke about manifestations of antenatal depressive symptoms in terms of cognitive characteristics (forgetfulness, ambivalence towards the baby and “*thinking too much*” [“*bizu bemaseb mechenanek*”], anger and ruminating on unattained expectations with feelings of guilt and anxiety) and somatic symptoms (reduced appetite, poor sleep) and certain behaviours (poor engagement with clinicians, difficulty bonding with their baby and refusal to breastfeed). Both women and HCWs commonly perceived depressive symptoms as a reaction (“thinking too much”) to social adversities such as the additional burden of pregnancy in the context of pre-existing poverty, marital conflict and perinatal physical health complications and they emphasized the difficulty that women with depression have relating with others, manifested in poor engagement with the clinicians. These findings are in keeping with other qualitative studies in LMICs [41, 42] and also among marginalised groups in HICs [43].

All three groups of respondents emphasised social attribution and somatic manifestation of depression, also seen across a range of other cultural settings [34, 44] and consistent with findings from several other LMICs [24, 41, 45]. Presentation of depressive symptoms with somatic complaints might explain higher rates of emergency healthcare visits [38], non-indicated medical investigations, increased likelihood of misdiagnosis and lower detection rates of depression in such settings [46–48]. HCWs highlighted that under-detection of antenatal depression may also arise from: (1) the clinical difficulty for non-specialist workers in distinguishing a normal response to social adversity from a depressive episode, and (2) overlap between symptoms of depression and normal symptoms of pregnancy, such as sleep problems and fatigue. But, the variation in service users’ presentation of depressive symptom as somatic complaints and HCWs’ characterization of depressive symptoms in cognitive and observable behaviour changes also explain lower detection rates in the study setting.

Persistent exposure to “thinking too much” about social adversities was perceived by all types of respondent to be a cause of mental health problems, but also as a non-stigmatizing and normal reaction to social adversities. This finding supports consistent associations between depression and social adversities [22, 23, 49] reported in previous studies. It is also consistent with a systematic synthesis where social factors adding to the burden of pregnancy [24, 42] were attributed to “thinking too much”, a construct understood as a cause of depressive symptoms and often a cluster of problems, associated with sleeplessness, reduced appetite, tiredness and difficulty doing day-to-day tasks ass reported in previous studies [45]. Nevertheless, both these social adversities (additional burden of pregnancy in the context of pre-existing poverty, marital conflict and perinatal physical health complications) and depressive symptoms were often attributed to spiritual causes, such as the work of the devil or evil spirits, indicating a complex relationship between attribution of social and supernatural causes.

Women and HCWs accepted life difficulties as triggers of depressive symptoms through thinking too much. The framing by Ethiopian HCWs of social adversity as an important trigger for depressive episodes may enhance their commitment to providing psychological interventions in accordance with women’s coping responses to everyday problems. Prayer, support systems and psychosocial advice were therapeutic interventions suggested as probes in the topic guides based on previous work by our group [25]. Evidence-based interventions such as problem-solving therapy (PST) [50] may therefore be particularly suited to reduce social adversities, overthinking and isolation associated with antenatal depression in this setting. Although adapted for use in several African countries with different primary care populations [27, 51, 52], PST has not yet been adapted for use in Ethiopia. In a recent meta-analysis, culturally adapted psychological interventions were found to be more effective [53]. Thus, the cultural, conceptual, and person-related facilitators and barriers to psychological interventions must be addressed in the design of psychological interventions for depression in new settings [53]. Some barriers and facilitators may affect implementation and uptake, while others may affect intervention processes and mechanisms [30, 54, 55].

Stakeholders identified a few potential facilitators for the acceptability of the intervention included the developing system of mental health training and care, positive attitudes of HCWs towards integrating mental health into primary care and women’s culture of seeking advice. Potential barriers were: a woman’s domestic and farm workloads, fears of being seen in public during pregnancy and limited primary care staffing. In previous studies, psychological interventions for depression in pregnancy were delivered at home and integrated within other healthcare programs [28, 53]. Our findings suggest that in this rural Ethiopian setting, integrating psychological interventions into existing ANC provision through PHC facilities is likely to be more acceptable to women and HCW stakeholders because of working closely with women in the confidential health centre setting. Participants valued confidentiality as a reason for choosing facility-based healthcare clinicians, rather than community based HEWs. Although the “friendship bench” has been a highly successful platform for delivery of problem-solving therapy in some African settings, concerns about stigma and the need for privacy favoured incorporating PST into routine ANC [27].

### Strengths and limitations

Strength of this study is the in-depth exploration of service user and providers’ perspectives across facility-based and community-based components of routine maternal care. However, the transferability of our study findings to other settings may be limited by the specific sociocultural context in this area of rural Ethiopia. Nonetheless, there is scope for transferability of the findings to other low-income, rural settings in Africa. Future researchers should consider further triangulation of results with the views of family members such as women’s husbands, who often share healthcare decision-making in this setting.

## Conclusions

Given the important impact of social adversity and situational stressors on psychological distress among pregnant women in this setting, psychological interventions that equip women with mechanisms for addressing real-world difficulties may be acceptable and appropriate for this population. Intervention design should accommodate the identified facilitators and barriers to implementation.

## Supplementary information

**Additional file 1: Supplementary file 1.** Interview Guides for women.

**Additional file 2: Supplementary file 2.** Interview Guides for Care Providers (midwives, nurses, health officers).

**Additional file 3: Supplementary file 3.** Health Extension Workers.

**Additional file 4: Supplementary file 4.** Code list and matrix of themes across cases.

## Data Availability

The datasets generated and/or analysed during the current study are not publicly available because consent was not obtained from participants for that purpose. The full anonymised dataset is, however, available from the corresponding author on reasonable request.

## Abbreviations

ANC: Antenatal Care
HCWs: Healthcare Workers
HEWs: Health Extension Workers
HICs: High Income Countries
IPV: Intimate Partner Violence
LMICs: Lower Middle Income Countries
MCH: Maternal and Child Health
PHQ-9: Patient Health Questionnaire-9
