# Supplementary material for: Stakeholder perspectives on antenatal depression and the potential for psychological intervention in rural Ethiopia: a qualitative study
Source: BMC Pregnancy Childbirth. 2020 Jun 22;20:371. doi: 10.1186/s12884-020-03069-6 (PMC7310345; doi:10.1186/s12884-020-03069-6)
Supplement: Supplementary file 1 — Additional file 1: Supplementary file 1. Interview Guides for women. [file 12884_2020_3069_MOESM1_ESM.docx]

## Supplementary file 1: Interview Guides for women

**PRIME Ethiopia: Topic guide for maternal mental health intervention development**

**For women who have experienced perinatal distress**

**Preamble:** There have been some mental health research projects working in this area for some years. We are speaking with different care providers and mothers themselves to help us understand how best to design a service for pregnant women and women in the first year after birth. Your opinions, ideas and concerns are very valuable to us.

As we have discussed, your answers will be kept private and no-one, besides the researchers, will be able to link your name or work location to your answers.

Thank you for your time in speaking with me.

1. What sorts of emotional difficulties do you think are faced by mothers during and after pregnancy in this area?
   - Can you describe how these emotional difficulties affect women? Their health? Their work?
   - How do women express these emotional difficulties? At home? To their friends?
   - How do women express emotional difficulties when they come to the health centre?
2. What sort of social problems do you think are faced by mothers during and after pregnancy in this area?
   - How do women cope with their social problems? What happens when they can’t cope with them?
   - How do social problems affect women’s emotional health? How does it affect their pregnancy and childbirth?
3. When you were pregnant/postnatal, you told our project worker about symptoms of feeling distressed. [*recap to the woman which symptoms she endorsed on the PHQ-9*]. Please tell me about how you were feeling at that time.

- What was the reason for you feeling distressed at that time?
- What did you do to feel better?
- Who did you tell about your problems?
- What help did you receive?
- How satisfied were you with the help you received? What additional help did you need?

1. What help do pregnant/postnatal women with emotional difficulties need?
2. Who do you think should provide the different types of help you have mentioned?
3. If not already mentioned, what do you think the different types of care providers should do for these mothers?
   1. Women’s development army/ health development army
   2. Health Extension Workers
   3. Nurses
   4. Midwives
   5. Health Officers
   6. Mental Health Nurses
   7. Doctors
   8. Priests
   9. Husbands
   10. Other family members
   11. Friends
   12. Other providers you can think of?
4. How could health centre workers help women with emotional difficulties?
5. How comfortable do you feel speaking to health workers about your emotional difficulties? What gets in the way of you talking to a health worker about your emotional difficulties? What would help to make you feel more comfortable?
6. In some other African countries, health workers help women with emotional problems by providing brief psychological support/counselling that focuses on how they can cope with their problems.

What do you think about this idea for Ethiopia?

- What do you think about the approach of helping women to cope with their problems? How helpful would that be?
- Who would be the best person to give the counselling?
- Where is the best place to do the counselling?
- What do you think about counselling being linked to antenatal care appointments?
- How long should each session be? How many sessions?
- Would it be better for each woman to have counselling on her own or in a group of women with similar problems?

1. What problems could there be with providing counselling to pregnant and postnatal women at the health centre?

- Time? Worried about confidentiality? Other approaches better (e.g. religious)? Idea that other people cannot help with problems?

1. What good side could there be with providing counselling to pregnant and postnatal women at the health centre?

**Ending:** Many thanks for your helpful and interesting answers.

Do you have any questions you would like to ask me?

Do you have anything you would like to say about what we have been speaking about?
