# Supplementary material for: Stakeholder perspectives on antenatal depression and the potential for psychological intervention in rural Ethiopia: a qualitative study
Source: BMC Pregnancy Childbirth. 2020 Jun 22;20:371. doi: 10.1186/s12884-020-03069-6 (PMC7310345; doi:10.1186/s12884-020-03069-6)
Supplement: Supplementary file 2 — Additional file 2: Supplementary file 2. Interview Guides for Care Providers (midwives, nurses, health officers). [file 12884_2020_3069_MOESM2_ESM.docx]

## Supplementary file 2: Interview Guides for Care Providers (midwives, nurses, health officers)

**PRIME Ethiopia: Topic guide for maternal mental health intervention development**

**For Care Providers (midwives, nurses, health officers)**

**Preamble:** There have been some mental health research projects working in this area for some years. We are speaking with different care providers and mothers themselves to help us understand how best to design a mental health service for pregnant women and women in the first year after birth. Your opinions, ideas and concerns are very valuable to us.

As we have discussed, your answers will be kept private and no-one, besides the researchers, will be able to link your name or work location to your answers.

Thank you for your time in speaking with me.

1. What sorts of emotional difficulties do you think are faced by mothers during and after pregnancy in this area?
   1. Can you describe how these emotional difficulties affect women? Their health? Their work?
   2. How do women express these emotional difficulties? At home? To their friends? How do women express emotional difficulties when they come to the health centre?
2. What sort of social problems do you think are faced by mothers during and after pregnancy in this area?
   1. How do women cope with their social problems? What happens when they can’t cope with them?
   2. How do social problems affect women’s emotional health? How does it affect their pregnancy and childbirth?
3. Can you describe what you have learned about depression in mothers during and after pregnancy?
   1. How often they occur?
   2. What are the features?
   3. How would you detect depression in pregnant and postnatal women?
   4. How would you help a woman with this condition?
4. Can you describe your experience, if any, of working with mothers with emotional problems or depression?
5. What sorts of help do you think mothers with emotional difficulties need?

(Leave open initially, then, if necessary probe for detail under following headings)

- 1. Help to address their social problems, e.g. poverty alleviation, addressing violence
  2. What do women with emotional difficulties need from health workers?
     1. Detection, opportunity to talk, information, support, medication, counselling?

1. Do you think all mothers should receive this help or only some mothers?
   1. If some mothers only, what sort of mothers? *(probe for risk factors)*
2. How do you notice emotional difficulties in women? How comfortable do you feel asking pregnant and postnatal women about their emotional health? What gets in the way of you asking women about emotional health?

1. What is your experience of using screening questionnaires to identify emotional problems? In some countries, pregnant and postnatal women are screened for emotional problems using questionnaires. What do you think about that approach? How would that work in Ethiopia?
2. Some studies from other African countries have shown that health workers can help women with emotional difficulties by providing brief psychological support/counselling that focuses on how they can cope with their everyday problems. What do you think about this idea for Ethiopia?

- How would that work here?
- Which health workers would be in the best position to provide psychological support/counselling?
- Where is the best place to do the counselling?
- How long should each session be? How many sessions?
- Would it be better to do for the individual woman or for a group?
- What training would you need to do the counselling?
- What support would you need to do the counselling?
- If you provided counselling, how would it affect your other work?

1. What problems could there be with providing counselling to pregnant and postnatal women?

- From the point of view of health workers?
- From the point of view of the women?

1. What advantages could there be with providing counselling to pregnant and postnatal women?

- From the point of view of health workers?
- From the point of view of the women?

**Ending:** Many thanks for your helpful and interesting answers.

Do you have any questions you would like to ask me?

Do you have anything you would like to say about what we have been speaking about?
