# Supplementary material for: Stakeholder perspectives on antenatal depression and the potential for psychological intervention in rural Ethiopia: a qualitative study
Source: BMC Pregnancy Childbirth. 2020 Jun 22;20:371. doi: 10.1186/s12884-020-03069-6 (PMC7310345; doi:10.1186/s12884-020-03069-6)
Supplement: Supplementary file 3 — Additional file 3: Supplementary file 3. Health Extension Workers. [file 12884_2020_3069_MOESM3_ESM.docx]

## Supplementary file 3: Interview Topic Guide Health Extension Workers

**Health Extension Workers: Engaging pregnant women to attend health facilities for care**

Thank you for agreeing to speak with us. We are interested to understand all about how HEWs work with pregnant women in order to help them visit health centres/facilities.

1. Can you please describe HEWs work with respect to getting pregnant womento attend/visit health centres/facilities?
2. What about the pregnant womanhelps them attend?

(*Probes: personality factors, social factors, family factors, practical factors, health factors)*

1. What about the pregnant woman creates problems for them attending?

*(probes: as for no. 3)*

1. Is there anything about HEWs may help to get the pregnant woman to attend? Please can you describe these factors?

*(probes: personality factors, educational background, personal background, supervision factors, social factors, work factors)*

1. Is there anything about HEWs that may prevent pregnant womenfrom attending? Please can you describe these factors?

(*probes: as for no. 4)*

1. Are there factors in the community that may help pregnant women attend?

(*probes: cultural or religious imperatives)*

1. Are there factors in the community that may prevent pregnant womenfrom attending?

(*probes: as for no. 6)*

1. What have you done when a woman has not attended antenatal care? Can you share some examples of how you or your colleagues have successfully managed to help women attend a health centre/facility where there were barriers to her attending?
2. Can you suggest some ways that HEWS can ensure more women attend a health centre/facility?

(*probes: content of engagement; the manner/quality of the engagement; the frequency of the engagement; the logistics of the engagement; the co-opting of other people to motivate)*

1. What sort of social problems do you think are faced by mothers during and after pregnancy in this area?
   1. How do women cope with their social problems? What happens when they can’t cope with them?
   2. How do social problems affect women’s emotional health? How does it affect their pregnancy and childbirth?
2. What sorts of emotional difficulties do you think are faced by mothers during and after pregnancy in this area?
   1. Can you describe how these emotional difficulties affect women? Their health? Their work?
   2. How do women express these emotional difficulties? At home? To their friends?
   3. How do women express emotional difficulties when they come to the health posts?
   4. What do women with emotional difficulties need from health extension workers, healthcare centre, or from the community?
   5. What type of treatment do women prefer for their emotional difficulties during their pregnancy (probe: drugs, holy water, traditional healers,..) why they preferred it?
   6. What are your thoughts about pregnant women receiving help for stress from the health centre/facility together with their antenatal care?[Probes: type of help like counselling, acceptability and feasibility on the side of mothers and providers, place of counselling and the number of sessions).
   7. If women were able to receive help for stress from the health centre/facility together with their physical health care, how would this affect your work? (*probes: Would HEWs need to do anything differently? How would these changes be perceived? What would HEWs need to help them do things differently as described by them?)*
3. Can you describe your experience, if any, of working with mothers with emotional problems or stress?
4. What sorts of help do you think mothers with stress or emotional difficulties need?

(Leave open initially, then, if necessary probe for detail under following headings)

- 1. Help to address their social problems, e.g. poverty alleviation, addressing violence
  2. What do women with stress or emotional difficulties need from health extension workers?

**Ending:** Many thanks for your helpful and interesting answers.

Do you have any questions you would like to ask me?

Do you have anything you would like to say about what we have been speaking about?
