# Supplementary material for: Stakeholder perspectives on antenatal depression and the potential for psychological intervention in rural Ethiopia: a qualitative study
Source: BMC Pregnancy Childbirth. 2020 Jun 22;20:371. doi: 10.1186/s12884-020-03069-6 (PMC7310345; doi:10.1186/s12884-020-03069-6)
Supplement: Supplementary file 4 — Additional file 4: Supplementary file 4. Code list and matrix of themes across cases. [file 12884_2020_3069_MOESM4_ESM.docx]

## Supplementary file 4: code list and matrix of themes across cases

| **Codes** | **Description** |
| --- | --- |
| depression impacts | women with depression get sleepy. they are not engaged at work and during ANC (women with depression are quiet and no concentration during ANC) |
| family involvement in intervention | midwife use routine letter to engage husbands to mediate IPV and so depression. Family (child, neighbour) is contacted to approach the mother for care |
| mhGAP training appreciated and used | |
| marital conflict as triggerer | problems at home or marital disharmony as a cause of depression |
|  | depressed women feel internalized stigma |
|  | women with psychosis come to delivery without carer |
| presentation of symptoms | women report depressive symptoms like being sleepy, want to be lonely to healthcare providers |
| Communications | midwives perceived by women as authority figures, staff need to oridinarily communicate in a local context |
| therapeutic alliance | being friendly, greeting, showing welcoming face, being close to help to women |
| poverty affects help seeking | women with extreme poverty do not come for help |
| alcohol triggers depression | alcohol drinking husbands expend money for alcohol, leave all workburden on women that women get overloaded and feel depressed |
| predentation of Sx, intervention | sleepyness' commonly reported as symptom to clinicians |
| lack of support/work burden | women with depressin even conceal their illness to report others due to lack of social support, need for subsist because of poverty |
| losing children triggers depression | giving child to others due to poverty, perinatal loss and memory of adversities during labor |
| marital conflict as triggerer | marital conflict raised as a cause for depression |
| Depression impacts engagement | women with depression do not look at the healthcare provder, look at the wall instead, get quiet |
| comfort to ask the symptoms | the healthcare providers' confort to ask the Sx o f depression |
| need manual | healthcare providers requested manual to screen or detect depresion |
| happy for the program | participants raised their happiness to be asked and to be part of the intervention program |
| potential providers of intervention | midwife nurses, HO and others are supposed to provide help |
| place of therapy | ANC ward, home, delivery ward |
| number of session | daily, weekly, biweekly, 3, 4 |
| group vs individual | women's and providers' preferences of therapy type (either group of individual) and the reasons of preferences |
| resource needed-training | resources needed to start the intervention (training, manual, |
| burden on clinical routines | staff already providing advice to women with depression, helping women as major responsibility; helping women does not add further clinical rouutine |
| staff work burden | risk of being staff burden on quality of intervention |
| acceptability | challenges related to acceptability of the intervention |
| advantage to staff | Tdvantage of starting the intervention to women |
| staff need supervision | The need for discussiong the cases faced by each healthcare worker in groups among the staff members |
| happy for the program | participants raised their happiness to be asked and to be part of the intervention program |
| Perinatal emotional problems are rare | the participant reports that no else women faced such recuring emotional problem except herself |
| CS impacted work and so depression | women complained their difficulty to work due to CS incesion |
| depression impactsa selfefficacy | a reflection of women's beliefs that they cannot do what their couterparts can do. |
| may God take my life | extreme poverty was a reason for women for being unable to take rest during their perinatal complications like CS incesion |
| told nobody | women simply worried and or prayed without telling the symptoms to people. MH Sx not reported to clinicians |
| waiting God's time | extreme poverty prohibited women to get mental healthcare that they are simply waiting the God's will to recover without any other treatment. |
| thinking too much | women perceived emotinal problems 'thinking too much' than as 'mental health or emotinal problems'. |
| losing children triggers depression | women who lost the child perinatally and those whose child has been taken by their relatives are worried of about the loss. |
| discontinued contraceptives | a woman with depression and with persistenet perinatal loss recommended to take contraceptive injection and to take follow up. But, she discontinued the contraceptive injection with no justifiable reason |
| forget everything and stop worrying | women forget worrying when they are with people |
| perinatal complications | women with perinatal commplications like CS and burning sensation during urination complained servere pain and lack of support to subsist her family |
| lack of support | postnatal woman complained lack of support from her husband for her perinatal complications and work to subsist the family |
| perception about postnatal pain | the belief that 'nobody else faces this type of postnatal pain in the community' |
| staff negligence | the staff in the delivery unit were perceived by the participant as careless and were attributed to be the reasons for the pregnancy loss of the mother. |
| pray to cope | prays to cope perinatal problems |
| Government support requested | women requested government support for their poverty alleviation |
| expected roles of husbands | roles or help that women expect from their husbands |
| expected roles of PHCs | roles or help that women expect from primary healthcare staff |
| expected roles of priests | priests help women by baptizing women with depression |
| expected roles of HEWs | roles or help that women expect from HEWs |
| expected roles of family | roles or help that women expect from family members |
| illness not accepted | woman with long complains about physical and mental health denied that her illness was not mental illness. She supposed it to be due to her child loss |
| session period | womens' preference for how long a session should be |
| adherence challenges | reflection of challengs and opportuniteis to adherence PST intervention schedules |
| benefit to mothers | women's understanding of psychosocial intervention benefits to themselves |
| poverty support focussed | women emphasized on the need for poverty support which may also enhance therapy |
| satisfaction- worker |  |
| satisfaction with advice |  |
| satisfaction with advice/counseling |  |
| confort to ask the symptoms |  |

Matrix of themes across cases

| **interviewee** | **coping** | **manifestations** | **triggers/causes** | **acceptability** | **Fidelity** | **relation with others** | **therapist skills/competence** | **intervention delivery** | **roles of others** | **opportunities and challenges** |
| --- | --- | --- | --- | --- | --- | --- | --- | --- | --- | --- |
| provider 001 |  | women with depression sleep most of time affecting thier work. They may not eat as expected. They do not work as their mind is busy. They tell us that they hate to meet people and alienate self. They have poor engagement. They turn fact ot opposite when you talk them. reasons for why: "feels sleepy, she has low appetite, I only sleep, I feel bored and yawning.." | problems at home like husband's drinking habit, workload where a a mother becomes solely responsible to care at home. Many wome associate with work. For example a women cried saying that she has no support and her child has been taken to Addis by the relative. children at home may also be disobedient and women will not be capable to get along with their husband. | after hours of discussion women forget everything when they are asked for reflection (do not listen). Sometimes they feel they were nagged. Provider here suggests not to be upset for such conditions where a woman forgets everything. Interviewee happy to get skill to help mothers promote mental welbeing. | P: It has no effect on me on the works that I do in this place, it is something related to here, that thing, after you finish asking, you register it, and there is nothing special about it. If I gave her counseling, there is nothing that would take up so much of my time for other works, since it is the thing I do, um, there is nothing that impacted me so far, um, well, it is the workers that do the registration and you can do that later on or the next day or you could even give it to the next shift taker when you go out, um, but I should take her ideas really well, since it is my job to do that, and since the counseling is needed by others as well and not only this there are other things. Counseling is a must, um, since that did happen it doesn’t have any other impact on me. P: That, sometimes, when there is work overload, there is a tendency to get fed up with the counseling, you get fed up, | women consider healthcare providers as authority figures. This is an opportunity for fair communication. them and discuss about their problems, mothers really see us as authority figures, but if you humble yourself and talk to them as ordinarily as possible with the language that they can understand, it can solve their problems, this | interviewee tellss that she is confident to ask the patients about the symptoms of depression. Women with psychological problems described as non-engaged and forgetful of what has been tol. This was described as a signal to detect depression. Being friendly and identifying the mothers suggested for open communication of mothers. the providers closeness to the mother maters a lot: understand needs of women, greet them at beginning, call her name properly, looking their face instead of focus on paper (L 310). warned that if no closeness women do not come again. using the language of women, women consider providers as authority figures. people in rural area look your face,. your approach matters, it should be friendly: greet them at the beginning, don't intrupt with phone calls, call her name properly. if you are doing something elles like writing while she talks, she will not be conformatalbe. | provider focused on telling/advising/educationg (mainly 'do this and don't this') women and the family. For example the provider tells a woman who cried for her child taken by relattives not to cry. contents include: advice on using pschiatric medications. midwife nureses are best to provide counseling since they are women and they CLOSELY work with mothers. Individual counseling than group counseling for privacy issues suggested. sessions: everyday | providers discussed problems of women with family (husband, children) ( 156-161). They used a letter to call the husband and to discuss family systme problems (183-189). | short of money (transport?). Short screening tool instead of opening mhGAP manual; further knowledge in the area for providers (training). If HEWs are not available on date of appointment they will not come again. Hospitality of the healthcare staff during delivery and good information and advocacy about the service to mothers. domestic chores preventing women to attend ANC. there are husbands who encourage their wives to attend to be checked. |
| provider 002 | women with low mood prefered loneliness and chose social exemption. They mostly choose to sit home alone and cry or go to churches and holy water than discussing the problems with people | causes abortion and so reproductive health challenges. Depresion reflected as Silence, cry and sadness (not happy during delivery). "because they don’t value people as such they won’t get close and talk about their problem that easily They come to the institute and take what they want but refuse to discuss their problem. In addition to this they have negative expectation from the society so they are mostly silent. They think people are going to do things to them". the Sx seen from women's face. | husband drinking habit and leaving all household burden on women as a trigger. Repeated birth while depending on the social support of the community as cause of depression. "If a mother gave birth repeatedly they will say things like why is she giving birth like a goat". on the other hand, her husband may not allow her to use family planning methods wanting more children | counseling prevents and creates insight to prevent the problems like reducing chance of suicide. it alos gives satisfaction to workers (456-463). But the other cases that I told you before will not come to us because they will consider their case as spirit or satan and didn’t tell to their families about their cases. We got this information from the cases who came to | P ? No it won’t because when a mother comes in for a visit you will ask her questions, you do that to save lives, you don’t have to think about other things, you shouldn’t. since you are there to save mother’s life you should spend time with her and not be worried about the time you spend there | when women have other types of diseases like STI, it is difficult for healthcare workers to communicate. They use informal ways to communicate like making the woman ask him to support her for treatment . Women with depression can be identified by looking their face and then asking questions what wrong is there with them. | the need to tell professional ethics related to confidentiality to engage women. Women do not start to tell their problems immediately. Once they start they tell their challenges at home, which the providers observed that they are not earting as their weight change shows. women's emotional problems can be observed from their face and we ask them what is wrong goes today and let them start talk. mothers not immediately asked about medical issues (asked about social, personal and envital issues) | finding ways and communicating with the husband to discuss about birth spacing, family planning and nutrition and caring pregnant women. Women ask abortin and we abort if it is less than three months. This makes them happier. The MH team provides counseling for women with MH problems. but, every nurse in a clinic is linked with maternal service that all should be trained on MH. separate room needed for counseling. 45' individual counseling in separate room | husband told called for clinical assesseement when discussion is needed or when a woman is diagnosed with STDs or HPV. | healthcare workers are already friendlly and there is well furnished room for mothers. there are problems on how to communicate the womens' challenges witht their husbands. Ex diagnosis results and conflicts. Having better service than before example rooms with curtain for ANC and delivery, coffee cermony. PHC open for improvement through direct feedback from mothers. 3-4 midwives on duty. so, the service itself attracts. "Yes they have understood it well. When we communicate about it, about seven animals have been slaughtered for a woman. " |
| provider 003 |  | Sx include: suicidality, forgeting, silence and self isolation thinking that they are not heard in the community. Tendency of magnifying small problems as serious one. | "women come to us when they have fight with him". poor standard of living | positive belief of the providers about advice/ psychotherapy. "I: What benefits would you say the counseling given for pregnant mothers has for women? P: I mean it serves them so that they will not harm themselves because sometimes they go to the extent of suicide when they are in worse condition so, if we can resolve their problems earlier, it will serve to prevent them from getting to their worse state. it gives us satisfaction". |  |  | P: I mean, when they describe it, it might depend on how close they feel, on the person who is close to them, now, in previous month, there is a mother we have followed up, every time she feels stressed we had followed up on her, so, when she describes her thoughts it was very clear, she was frank with us. Now, when she told us, she clarified on the time when she got into an argument, the ways that lead there, tells us just like a friend. women tell that they are stresed or you can see from their face or if stressed, they don't look at you and cannot attend what the worker says. do you feel comfortable asking them? P: If I see the symptoms on them, I ask them. | women with severe MH problems are discussed with health officer, a team for MH using mhGAP. 40' in health facility by close people to women for four sessions. |  | teenage pregnancy and conflict with family. R: especially life is very weak. In our area, life is just from hand-to-mouth. It is just becoming pregnant without something to taste. Providers percieved that the proposed intervention facilitates their existing work than being a burden for them. stigma from community when a woman goes back and forth to clinics |
| provider 004 |  | pregnant women should be happy, but such women are against self thinking suicide and sometimes fighting with their husbands. In presenting their symptoms, they do not have epereince of expressing themselves. "expressing themselves. When you notice something on their face you will ask them if there is any problem at home. and when you ask them like that, they just respond generally they did not tell you something specific. there is no habit of expressing clearly. You are the one who make them to talk more". depressed women may feel sad, hate to love their baby, refuse to breast feed. | fight and divorce with their husbands as a triger of minor depressive symptoms | positive belief of the providers about advice/ psychotherapy provided that there is training. Providers awareful of the unmet needs of women that need to be fullfilled. Workload raiseed as a challenge. Coulseling is helpful as it lets women share their cofidential issues that makes them worry. it also gives satisfaction to workers. | P: after all she is pregnant mother it is our duty to follow her health and help her to pass through the pregnancy with peace. so may be with respect to , if I spend 30 minutes or more with one mother and if there are another mothers waiting… because if there is another person who give the counseling, you just do your part and link her with that person then the work will be more facilitated but when you face such cases you might spend 30 to 45 minutes with one mother at that time the mother who is waiting outside may hustle. there might be situation like this otherwise, this is my job because treating that mother up to the end is my job but in relation to time it might have some impact |  | P: most of the time we did not encounter severe depression. after they gave birth. we do not face depression cases or any other psychological problems . but during their pregnancy they sometimes have feeling of "why would I get pregnant, I carry his baby and…"? there are some depressed. P: they got depressed they cry, sometimes they get tiered easily, they might feel tired when they cry and depressed too much if there are such symptom then I suspect for this problem. providers also used the mhGAP manual to detect but, by memorizing the Sx in the manual. good to ask the Sx before service in a form greeting and personal questions than asking at the end of service. clinicians happy to ask the Sx. | mild problems like wanting to be alone and cry with their husbands are common. But no report of severe Sx. Anyonne who has training and who is close to them and who can keep their privacy and has private office for counseling for 15-30 minites every 15 days. first psychoeducation is given and then medication if it goes worse by HOs. |  |  |
| provider 005 |  | such women hate self and to care themselves which leads other infections, increases poverty and maintains depression. They feel lonly and feel stigmatized. "when I ask them like “how is your social life with your neighborhood? do you feel lonely?” They say “Yes I feel. They usually feel lonely, feel sad without a reason, feel tired without a reason, and the like”. | polygamy and resulting reduced psychological support to a mother as trigger. "their husband might have two houses. I: what does it mean by two house? P: he might have two wives"; cultural beliefs in the postnatal period like leaving a mother alone | but women may get bored for repeated sessions. they may not come on appointment date. | P: well it might not have impact on me. It is the other way.  As you read more, you gain more knowledge. Thus if you work that thing side by side, I don’t think it will have that much burden on me |  | P: up to nine month, until she give birth she will gain one point five kilo. But when they constantly come monthly, fifty two, we ask her questions like “what is wrong? Don’t you eat a food your house? You have to gain this much weight.” and so on. They might tell you their problems. P: the people that come here are usually rural people. We do ask them about it. It don’t totally fulfils depression criteria but it somehow does partly for mild the depression.  P: Mostly I came across mild cases. I haven’t encounter a patient who starts a medication or that I made her start. clinicians do not ask wommen about depression (P: E I haven’t ever did such thing. E’ but from my experience if I have many patients I might not ask this thing. I only ask such questions only if there is no much patients or if I noticed something on her body. I haven’t ever asked patients with a prepared question). including Sx in partograph form??? | 30-1 hour in health posts |  |  |
| provider 006 | women go to religious places. Things that are done there will be done |  |  | cultural factors such as belief that women's not going out from home are widespread. Incase a woman has violated cultural beliefs, she might be stressed for that. "P: it is better to use the psychosocial education for treating patients rather than using medications". providers already engaged in providing psychoeduction but, complained about lack of training. women also do not go to referral centers since they do not consider it as an illness. if service is given here, we can serve them here without need of referring them. counseling reduces sucidality and gives the provider satisfaction | P: this wouldn’t affect my job right now, because our job here is to serve the public so the service we provide is identical for mothers and others this is not a different service, it may be that my skills are inadequate to provide the service so if I have additional capacity and serve mothers with it will not affect the job. there are some who doesn’t require it there should be a staff distribution for example instead of providing all related service at one room all services should have one dedicated room for the service like family planning, by having each rooms dedicated for a service and including the psychosocial education would make the service better if that is not possible we can still provide it right there so that the health problem wouldn’t be this much of a problem. |  | startign with social and personal issues suggeested and is practiced. mhGAP used to detect depression. Clinician happy to ask the Sx and to detect. Including the questions in the partogrpah suggested. | psycho-educaton then medication. But, currently psyco eduction is not complete due to lack of training on it though it is preferred to medication. So we are referring women for better service to hospitals though women do not likely attend it. |  |  |
| provider 007 | all psychiatric illnesses viewed as being possessed by evil spirit. People prefer holy water and 'dua prays' among muslims. Women with depression hide and isolate themselves. | such women isolate themselves and compalin sleep problems. she dropped her usual duty and was not able to socialize. | giving birth at early ages and worries on how to nurture, how to breast feed, worries whether the breast would suffice, ...; considering mental illness as without treatment. most live with mental illness or prefer traditonal treatments. | !What I think about it is, since the community don’t consider it as an illness, there is a less likely chance of visiting health centers". As a result, patients prefer religious places as treatment options or they live with the problem assuming that it is not treatable. patients are in pressure between the views of the community and the views of the healthcare providerrs about mental illness and its treatment. through counseling we can bring change in perception in the community. by doing so, we can promote service quality since mh service is not complete in many facilities. |  |  | depresion not asked but, only women who found accidentally may diagnosed with depression. Ex when they refuse to breast milk. which we have came across accidentally. Since the community does not believe that mental illness is an illness, and also doesn’t have the awareness to go for treatment even if the case is there, the society manages it by themselves or handles it traditionally or else she might still continue to suffer. P: - I worked on the case for four years. I have worked for four and half years and I have encountered one case. clinicians are not asking about depression is it is missed. | psycho-education is given followed by medication. But, since the number of such women is few, we attach such women with psychiatric nurses. Participant valued psyco education. Midwives and HEWs are right professonals for psyco education in every health centere. | advice is given to family (husband or childrenen) when needed. | well established maternal service in PHC compared to other wards, well established mental health system (PRIME). But, need training and the screening manuall |
| provider 008 |  | "P. Ok, the community may discriminate them by consideringas the patients brought the disease by themselves". Even there may be abortion | nearly when they reach for delivery. When we discussregarding this issue and when we relate it with the mentalhealth care, they will show some unusual behaviours likecrying and sitting at home. When we get such kinds of case, | But the other cases that I told you before will notcome to us because they will consider their case as spiritor satan and didn’t tell to their families about theircases. They take patients to religious places and to magicians. Benefits from the service to workers may create complains/dissatisfaction. P: - If I start providing the service to the mothers now,normally, if I am not assigned for another task, it doesn’tcreate any influence on me. It’s all about helping; it isthe same, helping these patients and other patients. Itdoesn’t create any influence on me. |  |  | I.How do you detect depression in mother? Tell me how youfirst detect depression in mothers.P. It may be from the behavior that she shows and there maybe family history in addition to her behavior. When I seeher, she may has abnormal feeling and it may help toidentify depressed mother. P. I didn’t specifically screen depression cases until now.P. I may ask her general questions and sometimes we may notask her because of time but if we see some thing we maydiscuss about it based on what they said. I may not ask herpurposely. | based on mhGAP training |  | P. They consider health professionals sometimes just nextto their Lord due to this they will tell us each and everything that they faced. shortage of manpower in PHC may be a challenge causing overburden and loss of interest on the workers. Difficult topography |
| Woman 001 |  | Sx presented as being stressed and strugling with oneself while attributing the cause to extreme poverty | the woman attributed her poor living condition to her antenatal depressive symptoms. For example, I am living a very despicable life…ehh… I don’t have a house, I don’t have a land…ehh…it’s very difficult to raise these my five children. Thus, I started to become stressed when I think of that…ehh… I think of everything, I am living a stressful life" | P. I think probably there may be shortage of human power. I mean if two staffs are assigned for this work, the rest will be very busy by the other activities in the delivery room for example there is delivery, family planning and antenatalcare services in the family health clinic. So, if |  | women tell their stress to their husband first | comfortably told her Sx to clinicians | P: yes. they said, “you don’t have to be like this, don’t stress out yourself, you get depressed when you think too much, you will be fine if you try to minimize your thoughts”, that’s what they advised me. Individual counseling one per 15 days in healhcare facility. |  |  |
| Woman 002 | women hads nothing to do except sufering and prayers: "I used to say, “ God, you are the one who created me, you can do anything on me”. I was hopeless. I used to say, I will be staying until God want me to stay alive. Everyone" women told their miseries to family, but family considered theier miseries as solutionless. | women present depressive symptoms as being stressed: "P: well, I told them that I feel mentally stressed...ehh… she considered her delivery complication as unique to her alone and they asked me, “ what makes you feel stressed”? then I replied that, it’s because I think about so many things…ehh… it’s because of the thoughts". they also present as physical Sx like 'worn out'. women presented symptoms as unique symptoms to themselves alone: "P: nobody experienced that type of problem in this area…ehh… its only me who faced that type of problem, a women with two perinatal losses". P: I just went to the clinic …I told nobody at that time…ehh…I was worried by myself. | I am afraid I might die because of the illness. They told me not to do heavy work. But, I am doing it because I need. Loss of two children was attributed to the illness., communication of bakd news such as death of infant, surgical producedures during delivery and lack of support from her husband. Lack of rest to earn and prepare food stuff for daily subsistence. "They [clinicians advised me to take rest, but, I don't have anything at my home. Woman worked lonng hours despite her illness that made her feel pain in the night. If I can work hard to raise him now, he will support me when he grows up but God didn’t want that to happen, and I lost both of them." P: it had a burning sensation when I had the surgery. Ehh… it burned me a lot when I was trying to urinate. I wasn’t able to defecate at all | women with depresive symnptoms do not accept their symnptomms as emotional problem. "Sometimes I wish may God take my life". vs "I didn’t have emotional problem…ehh… I was used to think what would i eat after giving birth, how am I going to live, I can’t work having my child…ehh… because it’s difficult to take care of the child until he grows up" she linked the Sx to perinatal loss. "... you told me earlier that you felt like running away, you used to feel stressed … P: that’s because I lost my children. But, I didn’t have emotional problem before. I had been hurt when i gave birth …I was injured" |  | a mother's parents usually make decision for her. In the context of collectivist cultures, decisons are shared. Collective interventions than individual i nterventions may work??? The way providers tell the bad news to mothers may triger depression. "the child is passed you can't do anything". P: they didn’t try anything. They just said, “ try to wait Gods time, stop doing the work”. But, how could I survive if I quit working? I don’t have anything at home. I just need to live like this until God helps me to get through this. Or, I may go back to my families leaving my own life here…but, for how long should I have to stay with my families…ehh… I just try to survive like this until God allows me to live |  | individual counseling by healthcare workers every week. | good if PHC can follow us once they refer and good if they can service politely understanding patients. | P: yes, I feel pain but I don’t tell that to people. Because, I will lose my job…ehh… so, because of that, I wake up in the morning, eat something, have my coffee and go to work. I spend the day working and get back home. But, I feel too much pain in the whole night. household chores, mourning and some incidental activities might affect women's adherence to treatment schedules. |
| woman 003 | prayers and holy water were coping mechnisms among women since depression was attributed to God's will and superme power. "I ask God what I have done…all these suffering on me, you God be praised, it is your will I said and return myself" | the woman presented her Sx as "I was mentally suffocated". She considered pregnancy complication as unique problem to her alone. Depression affect dieting. Described the Sx as feeling stressed: "I told them [health workders] that I felt stressed…". | child loss aggravated the Sx. A woman lost three children commplained family conflict | women change their mind when there are some incidents. That might doubt their adherence. |  | child loss aggravated the Sx. A woman lost three children commplained family conflict. She was stressed due to communication of the healthcare workeers that didn't connsider her poverty. |  |  |  |  |
| Woman 004 | " I try to take out of the situation and try to do other stuffs or go to my neighbor’s or friend’s house when I feel like this that is it." | woman with psychotic Sx like buring cloth. "P: - Yes, I feel annoyed when I see some household items. Then, I started to take all the uninteresting household items out and hid it all. Then I got sick. When I got sick, I went to take holly water during my pregnancy." | depression comes from poverty. A woman gets angrier when she has no money to buy food stuff. Unfulfilled needs as trigers |  |  |  | health post |  |  |  |
| Woman 005 | "I feel better when I have fun and when I chat with people. Then, it started all over again within two or three days. Then, I got sick again" | "I feel like my head is tense…then I get stressed. I couldn’t able to sleep. When I have a headache, then, I physically get exhausted, I don't feel good when talking with peoole". "Thus, I don’t want to meet or converse with people. I just want to stay at home. I just stay at home and try to sleep if I can sleep otherwise, I just walk around…there is nothing else" | getting upset with something (children at home not her own and conflict with husband and death of mother) as triger. P: I didn’t face any problem. My illness is from God and nobody has done anything to me. Huaband insults, hits and drinks. Thus attrib uted to her illness,. | But, this year, I started taking this medication as I am getting sick. Then, I discontinued it after a while. It gets worse. |  | P: I told them that I am sick, I feel mentally stressed…ehh… I am not aware of myself… I told them like that and they asked me, “why are you feeling like this every day”? I said, I don’t know anything, I am feeling very stressed…that’s what i told the health professionals | they show us the risk and tell us the solutions like birth spacing. |  |  |  |
| Woman 006 | healthcare professionas were preferred to be consulted than neighbouring women for confidentiality, to reduce stigma and discrimination. "P. If I tell the women as I have mental health problem, they will discuss about me with other persons behind me.They didn’t keep my secrate and they may discriminate me" | Sx described as normal Sx o f pregnancy like vomiting, appetite changes and hating people: "I didn’t sleep many times but I feel tired, when I sit at one place, I want to sit many timeand unable to stand soon". " don’t want to meet other women and other women willnot meet me in this village". P. I don’t know the reason why but I want to live alone.P. I will assume as other people talk about me. P. I think as they will talk and gossip about me andbecause of this I want to live alone and I don’t want torent my houses and I am living alone. If I rent my house, Idon’t want to communicate with people. |  | P. I choose health professionals.P. The health professionals will give you good consultationand will tell you things that you should do but if they arethe women, they will talk about you behind you with thevillagers and they didn’t keep our secrate. |  | some women tell their symptoms to nobody. P. For nobody but there was one woman who went to Saudi | P. I didn’t feel stress when I tell them. I am free when Ispeak about my problem with the health professionals. |  |  |  |
| Woman 007 |  | women feel discriminated by the neighbourers | unplanned pregnnacy | P: She will get big lesson from that advice |  |  |  |  |  |  |
| Woman 008 | community support helps me attain my goals/plans. That makes me happy. I didn't go church, b/c "control myself so I don’t go there. They don’t hear what I am saying and I don’t hear what they are saying. In fact I don’t hear their praying. At that time I don’t know". family and community support helps women cope with their social problems. THINKING TOO MUCH | presented as "stressed with unknown reason probably thinking too much" and having headache/feeling of burn in the forehead/ and death wish. | severe sickness of her husband along with poverty and early childbirth created pressure of how to care | the woman valued advice than money. But, she complained priests for not hearing her and she was not also hearing them. Some persons do not want to go to hospital b/c they don't want to take medication. Women fear being rumored | HEW who was asked about challenges of providing therapy: "I don’t think it would create any problem. Instead, think it encourages our work and helps mothers with such problems recover from problems. it is also our duty. We as well as the health centre are working on the chain of maternal health. We also work a lot there. | P? I can’t. I was stressed out by saying "How could I tell this". I did not tell. People just know my condition without me saying" I am sick and stressed". Because I don’t want to stress out other person. I say I will try my best without telling to anyone. That make me stressed more. Most of the time I did not want to bother anyone. his/her problem, or part of it, they can’t be helped. Some people come and only tell part of their problem to get pills or some kind of medicine for temporary relief. | health centere |  | Prriests do not hear what i am saying and I don't hear them of course. |  |
| HEW REC 008 |  | Pregnancy increases poor women's overload and so makes them think too much, feel angry and devote time thinking and ruminating than working. "so, their mind will be busy ruminating ideas. they only think about it and ruminate". Women present the symptoms as' tension'. "say “I get tensioned when I hear omething, or when I hear children crying at home, or when he speaks”. | unmet needs such as being unable to feed family as trigers of depression. No attetion on the side of husband and community to women's mental illness. "R: yes, he was not working. He was insulting when the issue of work is raised. He makes his time pass in others’ house. Anyways she was telling me presence of high load in her mind and presence of three children who are nurtured by herself with the poor economy.". she was ruminating. women associate the illness with sata or evil spirit and or thinking too much. illnesses. They also associate mental illness with spirit or being bewitched or link it with other bad (amliko) that they prefer holy water, I think. but, prefered modern treatment for non-mental illness . "R: for example, when we go there to provide home to home service, there is nothing. Her husband is working being employed in another house. He comes to his home always only in the night. There are two kids in the house. Since they cannot manage themselves, there was no one at home to fetch water and prepares food for the kids. Emmm... because of this the mothers thinks a lot". | associate with satan, bewichment or spirit and go to holy water and women preferred holy water most of the time for mental illness while they prefer modern treatment for physical illness (943-946). B/c they know modern treatment is efective for physical illness like malaria. | good reception and family like approach is valued |  | health care providers need rapport establishement skills to improve acceptability of invervention. Emm... if there is no good reception, for example, like nagging them, mothers would not come again. The way women are asked maters whether mothers tell the symptoms. for example asking it as part of the healthcare. Starting from greeting, when we start exchange of ideas about her children, work, etc, she tells us clearly without hiding anything. To raise about this issue however, there should be another issue to link with or like utilization of family planning which she is following up or which she may also has discontinued. That is something that links with us. | HEWs coordinated the neighbourers and the husband for help. Women told to cosult PHC for any symptoms of complications. They are given a pumplet about 16 packages of health exxtension. These paper includes dangerous symptoms observed on a woman during pregnancy, delivery and at post-natal stage, nutrition, child care, how to feed a child and a pregnant women, the use of vaccination, family planning, how to use toilet, sanitation and hygiene etc. so, women are made to percieve the negative consequence of complications. | HEWs identify pregnant in colaboration with HDA and advocate them for ANC then refer them to PHC for ANC1. the rest of the ANC may back to HEWs. 30 neighbouring mothers organzzed to discuss health issues. HDA leaders send women. HEWs support to HDA includes: identify pregnant women, advocating adherence to ANC (by giving professional description), educating complications FP, vacination and recieving feedback from mothers. women and HDA leader use informal communiction during identification of pregnant women. ex " `i am looking you being different” what is new about?" postnatal care is done in PHC for women deliveing in PHC. we follow these women using information wrtten from PHC about delivery. mothers expect us make them forget their ruminating thoughts. |  |
| HEW REC 009 |  |  |  |  |  |  |  |  | women trained about maternal care services |  |
| HEW REC 010 | mostly women prefer to use cultural medicine. They prefer holy water with the belief that holy water makes them recover from their mental illness though that is not the only belief for them. |  | reduced capability to earn income during pregnancy as a cause of depression | since they are living toghetr, they listen to leader of development group. "Int.: Ihhh... are they more acceptable than you? Resp.: Yes, yes they are more acceptable.". Resp.: Most of the time, they prefer to use cultural medicine for mental illness. Besides Ihhh.... they usually go to holly water. | Resp.: Of course, the work load may increase. Indeed, it makes me busy as it is additional work. Therefore, I think it is burden for me. Resp.: Of course yes. However, I can do it as our purpose of coming here is helping our society. It may be compensated by our mental satisfaction. When a person who is severely sick is recovered from his sickness because of my support, I will be very glad and get mental satisfaction. | they usually tell for their family who are living with her. If the problem is beyond the family, they may tell for the neighbors or for us. when women are depressed, their previous behaviors changes. For example she may get quiet when I ask her. |  | women told the importance of getting maternal service for herself and for the newborn. Advised not to be far from the health center around time of birth. The importance of counseling the family and the husvand about the management of pregnant women was suggested. | HEWs identify pregnant women, encourage them to attend ANC and monthly conference, eductate them about the packages and vaccination, delivery, ANC. HEWs as intermediate workers between women and PHC representing women at PHC and advocating, following up and eductiong women to use PHC when they are with women. network with command post (teachers , HEWs and agri people). the greeting the a HEW gives to a woman sshould be consistent. it should not be limitted only to pregnancy. | lack of finance for trasnportation and cultrual factors about going out after delivery or during pregnancy. They also believe that a women should not get fresh air, light or heat during labor and were not given water nor food. Current mothers are mostly literate that is opportunity. women restricted from social activites at 7-9 months of pregnancy for not be labelled 'ayen awuta'. distance to healthcre and topograhy, husband sometimes are barriers. |
| HEW REC 011 | women preferred HEWs' advice since they get immediate advice whenever needed. "us everything clearly. Even though we advise them to tell everything for health center workers, they may not tell them what they tell us. This happens as we always communicate with them freely" |  |  | Resp.: Most of the time, they prefer our advice. They do not compare our advice with others. They ask us a lot of questions and we respond them freely. Eventhough we advise them to tell everthing to PHC workers, they do not tell them what they tell us. |  | Resp.: For their intimate friends. Int.: What do the expression "I am tied" and I cannot move freely as before mean? They do not conceal any secret for me. Even they tell me more than they tell their husbands. |  |  |  | low income and having HIV are barriers. Giving refreshement like tea may encourage women. Sometimes mothers may get hurry for market, or travel to home, etc.mothers fear being diagnosed with another disease. They doubt as if we do another diagnosis. |
| HEW REC 012 | "they [women] accept most ours. As you said, they can be advised by different persons. However, they come to us to confirm. When the advice givensomewhere is consistent to what we give them, they accept as if it is right. R: it is must that they find us first in the community" |  | unfullfiled childhood needs due to strict parental control like marrying whom one wants. Womans alcohol drinking which was supposed to be coping mechanism for unemployment was triger of depressive Sx. Hasband's drinking habit. "Emmm.. when they always show different behaviors... what Iwould say... when the husbandcomes home mad after drinking, she also tries to come homemad after drinking for revenge." poor marital r/ships like jeoleosy | women preferred HEWs for advice. "they accept most ours. As you said, they can beadvised by different persons. However,they come to us to confirm". interviewee openion: "Above medication I think advice isimportant". Consistency of advice b/n HEWs and PHC suggested | I think would be good. Themidwives tell them the advantagesand limitations of issues that benefits them. When thesemothers comeback to us, we will hearthe replica. They tell us these and these, ... the nursein the health center..... |  |  | counseling aims to raise awareness about adv and disad of maternal service. women attending ANC and who delivered in PHC are given a paper that explains their ANC status and the whereabout of delivery. 30' counseling for three days in health post (home suggested as not convinient at it may cause stigma). HEWs report what they did with the HDA and what they teach to the mothers. combination of both individual and group counseling suggested. HEWs make home to home visits to gather information about women's ANC status. they also get report from PHC about ANC and delivery. HEWs are best options | focus on prevention. HEWs need to be intimate, respectful without condiction and honest to women and has ability to understand individual difference. |  |
| HEW REC 014 | for confidentiality reasons, they prefer us. sometimes they warn us not to tell their secrets to others. | pregnancy before marriage as a risk factor for tension due to fear of disclosing to family while tension causes abortion. Even her opposite sex friend may not accept i t. | jeoleosy or marital disharmony as a source of women;s worries | women preferred health professional advice for confidentiality. Good if both povide the counseling service. | I. Does doing such kinds of tasks make you busy? R. It is our duty. Our duty is surveying those women who have psychological problem and stressed ones, raising awareness how to solve the problems and send them to hospital. |  |  |  | create good perception about healthcare system by telling its importance and the type of services given. It is motivating women utilie healthcare services | social activies like funeral and festivals |
| HEW REC 015 |  |  |  | wommen accept the advice, but some of them may not immplement it. |  | R: they tell us first to hide their secret. Meaning for their privacy purposes. Seond they assume that health extension workers would solve their challenges. HEWs asked the women when they observe women not caring thermselves or when their weight of the weight of the fetus reduces. |  |  | same network described as others | they may not want to go somewhere during pregnancy and the husband may tesion her for doing so. |
